# Fibrous Scaffolds From Elastin-Based Materials

**DOI:** 10.3389/fbioe.2021.652384

**Published:** 2021-07-16

**Authors:** Jose Carlos Rodriguez-Cabello, Israel Gonzalez De Torre, Miguel González-Pérez, Fernando González-Pérez, Irene Montequi

**Affiliations:** ^1^BIOFORGE, University of Valladolid, Valladolid, Spain; ^2^Center for Biomedical Research in the Network in Bioengineering, Biomaterials and Nanomedicine (CIBER-BBN), Madrid, Spain

**Keywords:** elastin like recombinamers, tropoelastin, fibers, tissue engineering, processing techniques

## Abstract

Current cutting-edge strategies in biomaterials science are focused on mimicking the design of natural systems which, over millions of years, have evolved to exhibit extraordinary properties. Based on this premise, one of the most challenging tasks is to imitate the natural extracellular matrix (ECM), due to its ubiquitous character and its crucial role in tissue integrity. The anisotropic fibrillar architecture of the ECM has been reported to have a significant influence on cell behaviour and function. A new paradigm that pivots around the idea of incorporating biomechanical and biomolecular cues into the design of biomaterials and systems for biomedical applications has emerged in recent years. Indeed, current trends in materials science address the development of innovative biomaterials that include the dynamics, biochemistry and structural features of the native ECM. In this context, one of the most actively studied biomaterials for tissue engineering and regenerative medicine applications are nanofiber-based scaffolds. Herein we provide a broad overview of the current status, challenges, manufacturing methods and applications of nanofibers based on elastin-based materials. Starting from an introduction to elastin as an inspiring fibrous protein, as well as to the natural and synthetic elastin-based biomaterials employed to meet the challenge of developing ECM-mimicking nanofibrous-based scaffolds, this review will follow with a description of the leading strategies currently employed in nanofibrous systems production, which in the case of elastin-based materials are mainly focused on supramolecular self-assembly mechanisms and the use of advanced manufacturing technologies. Thus, we will explore the tendency of elastin-based materials to form intrinsic fibers, and the self-assembly mechanisms involved. We will describe the function and self-assembly mechanisms of silk-like motifs, antimicrobial peptides and leucine zippers when incorporated into the backbone of the elastin-based biomaterial. Advanced polymer-processing technologies, such as electrospinning and additive manufacturing, as well as their specific features, will be presented and reviewed for the specific case of elastin-based nanofiber manufacture. Finally, we will present our perspectives and outlook on the current challenges facing the development of nanofibrous ECM-mimicking scaffolds based on elastin and elastin-like biomaterials, as well as future trends in nanofabrication and applications.

## Introduction

Current cutting-edge strategies in biomaterials science are focused on mimicking the design of natural systems which, over millions of years, have evolved to exhibit extraordinary properties (Zhao et al., 2013). Based on this premise, one of the most challenging tasks in the field of tissue engineering and regenerative medicine is the development of biomaterials and scaffolds that imitate the natural extracellular matrix (ECM), due to its ubiquitous character and its crucial role in tissue integrity.

The ECM consists of a complex mixture of interconnected molecules secreted by cells that arrange to provide a physical scaffolding for cells and tissues and promote physicochemical cues for normal tissue morphogenesis, differentiation, homeostasis and healing (Frantz et al., 2010; McKee et al., 2019). ECM-mimicking scaffolds should therefore be able to reproduce the reciprocal interaction between cells and the ECM, which involves certain bio-functionalities such as cell adhesion, protease sensitivity and cytokine release, amongst others. This complex interaction also involves physical and morphological features and is not restricted exclusively to biological signals.

The anisotropic fibrillar architecture of the ECM has been reported to have a significant influence on cell behavior and function. As a consequence of the intimate relationship between the cytoskeleton and the ECM, cells are able to sense and respond to the mechanical properties of the surrounding tissue by converting mechanical inputs into chemical signals (Galbraith and Sheetz, 1998; Geiger et al., 2001). Moreover, it is known that the fibrillar structure of the matrix components causes adhesion ligand clustering, which clearly alters cell adhesion and motility (Maheshwari et al., 2000).

From a structural point of view, the natural ECM comprises protein fibrils and fibers interwoven within a hydrated network of glycosaminoglycan chains, which endows it with a fibrillar and viscoelastic character. Fibrous proteins include collagens and elastin, with the former being the most abundant and main structural component of the interstitial ECM (Gordon and Hahn, 2010; McKee et al., 2019). Elastin associates with collagen and provides complementary function, mainly as elastic fibers. It consists of tropoelastin and microfibrils (Wise and Weiss, 2009) and is responsible for the reversible extensibility (recoil properties) of tissue. Elastin is particularly abundant in tissues and organs that require elasticity or undergo cycles of elongation and shrinkage, such as the lungs [3-7%, skin (2-3%), blood vessels (28-32%), and elastic ligaments (50%)] (Uitto, 1979). Moreover, it plays a key role in the functionality of these tissues due to its involvement in many cell-signaling processes (Almine et al., 2010, 2012). In this review, we will focus our attention on fibrous assemblies of elastin and elastin-based biomaterials.

A new paradigm that pivots around the idea of incorporating both biomechanical and biomolecular cues into the design of biomaterials and systems for biomedical applications has emerged in recent years. Indeed, current trends in materials science address the development of innovative biomaterials that include the dynamics, biochemistry and structural features of the native ECM (Acosta et al., 2020a). In this context, one of the most actively studied biomaterials for tissue engineering and regenerative medicine applications are nanofiber-based scaffolds. Several advanced strategies have emerged with the aim of copying the intricate fibrillar architecture of the ECM components and manufacturing biomaterials and matrices with a similar structure. These strategies are mainly based on our understanding of supramolecular self-assembly mechanisms to form nanofibers *in situ* and in the recent progress made in polymer-processing technologies. The way these nanofiber-based scaffolds are manufactured may change their nanostructure and, hence, their function and properties.

Nanofibers have unique properties that are mostly associated with a reduction in their diameter to the nanoscale, which translates into a significant increase in the surface-to-volume ratio and greatly affects the chemical and biological reactivity, as well as the electronic and mechanical properties, of the fibers (Ko and Wan, 2014). Given their high specific surface area and porosity, nanofiber-based scaffolds exhibit an extraordinary loading capacity for biological substances and active ingredients. Moreover, these nanofibers form an interconnected network of micropores that perfectly imitates the topographical features of the natural ECM and, as a consequence, comprise a suitable scaffold for cellular growth, proliferation and differentiation (Kenry and Lim, 2017). Consequently, rational design principles should be proposed to control fiber diameters and morphology, as well as pore size, to ensure that they are compatible with the cellular processes involved in migration through the ECM (Friedl, 2004).

Since the ECM is highly heterogeneous and there are important variations between different tissues, the nature of the biomaterials employed, the method for manufacturing the nanofiber-based scaffold, and its morphological features depend closely on the desired application. Thus, materials from natural sources are widely used because of their physicochemical and mechanical properties as well as their inherent biological recognition properties, which includes the presence of receptor-binding ligands and a sensitivity to cell-triggered proteolytic degradation. Nevertheless, natural materials have some important drawbacks, such as immunogenicity and pathogen transmission issues, and have a predetermined structure that limits the possibility of functionalizing them (Acosta et al., 2020a). Most of these limitations can be overcome using recombinant protein expression technologies, which allow the production of materials with strict control of their composition and physicochemical properties and, therefore, the production of tailored scaffolds with tuneable bioactivities. In this regard, genetically engineered biomaterials based on structural proteins have emerged as outstanding candidates for the production of ECM-inspired scaffolds. In this case, a wide variety of functional building blocks, including structural, self-assembling and bioactive domains, can be merged together using molecular biology techniques, thus opening up a wide range of possibilities in the production of protein-based ECM-inspired scaffolds (Acosta et al., 2020a).

Herein we provide a broad overview of the current status, challenges, manufacturing methods and applications of nanofibers based on elastin and elastin-like biomaterials. We will start with an introduction to elastin as an inspiring fibrous protein, as well as to the natural and synthetic elastin-based biomaterials employed to meet the challenge of developing ECM-mimicking nanofibrous-based scaffolds. This will be followed by a description of the leading strategies currently employed in nanofibrous systems production, which in the case of elastin-based materials are mainly focused on supramolecular self-assembly mechanisms and the use of advanced manufacturing technologies. Thus, we will explore the tendency of elastin-based materials to form intrinsic fibers, and the self-assembly mechanisms involved. Moreover, we will address other self-assembled motifs found in nature, such as coiled-coiled, β-sheets, and β-hairpin structures which, in combination with elastin domains, act as promoters in the formation of amyloid fibers. More specifically, we will describe the function and self-assembly mechanisms of silk-like motifs, antimicrobial peptides and leucine zippers when incorporated into the backbone of the elastin-based biomaterial. Advanced polymer-processing technologies, such as electrospinning and additive manufacturing, as well as their specific features, will be presented and reviewed for the specific case of elastin-based nanofiber manufacture. Finally, we will present our perspectives and outlook on the current challenges facing the development of nanofibrous ECM-mimicking scaffolds based on elastin and elastin-like biomaterials, as well as future trends in nanofabrication and applications.

## Elastin-Based Biomaterials

### Tropoelastin

Elastin is a fibrous and insoluble protein formed by crosslinking of its soluble precursor tropoelastin mediated by lysyl oxidase (LOX) enzymes (Brown-Augsburger et al., 1995). In nature, elastic fiber assembly starts with the production of tropoelastin in elastogenic cells. This tropoelastin is then transferred to the cell surface, where it clumps with glycosaminoglycans and accumulates as elastin aggregates. Therefore, since elastin is formed from tropoelastin, a detailed description of its properties is summarized below.

Tropoelastin is a 60-72 kDa protein comprising 750 to 800 amino acids (Brown-Augsburger et al., 1995). Its amino acid sequence is characterized by the alternation of hydrophobic and hydrophilic domains (Wise and Weiss, 2009). The former mainly comprise the non-polar amino acids glycine (G), proline (P), valine (V), and alanine (A). The hydrophilic domains are characterized by a high alanine (A) and lysine (K) content that promotes intra- and intermolecular crosslinking. Secreted tropoelastin normally have around 40 lysine residues, of which approximately 35 are modified by LOX-mediated crosslinking. The high degree of crosslinking confers an extraordinary stability on mature elastin and a very low turnover, which in turn can be considered to be why elastin lasts for the entire lifespan of the host (Hinek, 1997). Moreover, this crosslinking makes tropoelastin highly insoluble, thus preventing it from being manipulated and making it suitable for tissue engineering and other biomedical applications (Almine et al., 2010; Ozsvar et al., 2021).

**FIGURE 1:**
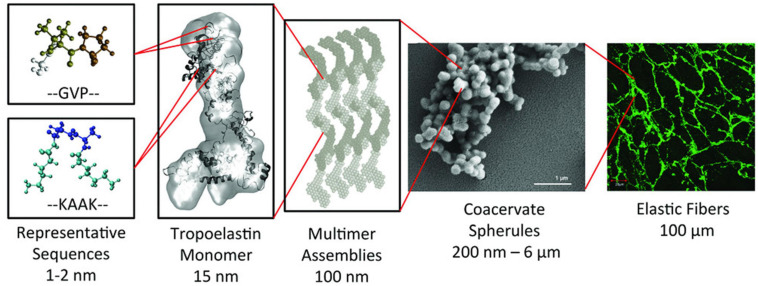
Synopsis of the hierarchical structure of elastic fibers. The monomer of tropoelastin is composed of alternating GVP-rich hydrophobic domains and hydrophilic K-containing cross-linking domains. This monomer assembles into multimer that evolves into spherical aggregate structures. These spherules will form a microfibrillar scaffold to form an elastic fiber. Reproduced with permission from (doi.org/10.1002/mabi.201800250), copyright 2019.

The alternating hydrophobic and hydrophilic domains in the structure of tropoelastin confer elasticity and thermal responsiveness on the molecule (Wise and Weiss, 2009). Thus, below its transition temperature (Tt), tropoelastin remains as a monomer with an elongated conformation, whereas when the temperature rises above Tt, the hydrophobic domains of the molecule start to establish weak interactions, thus giving rise to spheroid aggregates with a diameter of around 1 to 2 μm, which subsequently coacervate to form aggregates of up to 6 μm. This process is reversible, such that when cooled below Tt, the system evolves to obtain monomers again. Nevertheless, if the temperature is maintained above Tt, the coacervates firstly undergo a maturation process, then collapse and crosslink with LOX to give rise to branched fibrillar structures (Mithieux et al., 2009). This is a complex non-reversible process that is governed by several factors, such as the number and position of the hydrophobic and hydrophilic domains, pH and ionic strength, amongst others (Kaibara et al., 1996; Vrhovski and Weiss, 1998; Wu et al., 1999; Bellingham et al., 2001; Pepe et al., 2008) (See Figure 1). As a consequence of the complexity of this process, the formation of tropoelastin fibers or hydrogels will require extreme reaction conditions (high pH) and/or the use of harmful chemical reagents, such as organic crosslinkers (Mithieux et al., 2009).

### Elastin-Like Recombinamers (ELRs)

One interesting group of elastin-based biomaterials are elastin-like polymers (ELPs) and, especially, their recombinant versions (ELRs). These are protein-like materials whose composition is based on the repetition of conserved motifs found in the hydrophobic domains of native tropoelastin (Foster et al., 1973). Both ELPs and ELRs refer to the same kind of polypeptide, with differences in the production process.

**FIGURE 2:**
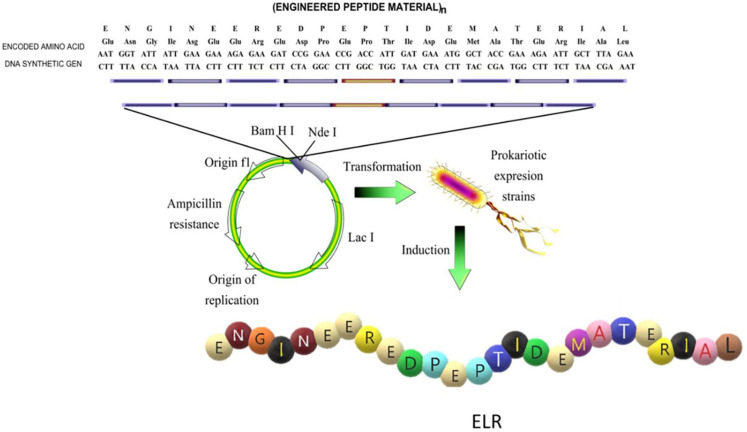
Schematic representation of the design of the sequence and production of an elastin- like recombinamer (ELR). Reproduced with permission from (doi.org/10.1016/j.addr.2018.03.003), copyright 2018.

The chemical synthesis of ELPs is a very long process involving a high number of steps and chemical reactions as well as the use of diverse precursors and solvents. Although it has been successfully performed for short ELP chains (Urry and Williams, 1985; Urry, 1988), high polydispersity was reported to be an important drawback when this synthetic strategy was applied to the production of larger or more complex elastin-like chains (McPherson et al., 1992). Recombinant DNA technology (Figure 2), which is based on use of state-of-the-art techniques in molecular biology to obtain cell factories, allows the production of advanced, complex and monodisperse biomaterials with absolute control over their composition. This technology opens up the possibility of producing a large number of protein polymers exhibiting any property found in nature, as well as other functions that are not present in living beings but which could be of particular technological interest. This method was first described by Conticello and coworkers for the production of ELRs (McMillan et al., 1999), and nowadays it is well-known that this technology allows the production of monodisperse ELR chains with the desired amino acid sequence and physical properties (Urry et al., 1985; Martín et al., 2010).

The amino acid sequence of ELRs generally comprises repeats of the (VPGXG) pentapeptide, where X is any amino acid except proline. All functional ELRs present a reversible LCST (lower critical solution temperature) phase transition (Urry, 1993). This means that in aqueous medium, and below the transition temperature (Tt), the polymer chain remains soluble, whereas above this temperature the ELR chains fold hydrophobically into a β-turn (Tamburro et al., 1990; Urry, 1993) and undergo a conformational transition, thus leading to phase separation (Urry et al., 1985; Manno et al., 2001). It has been shown that the LCST of ELRs depends markedly on the amino acid sequence and that, as a consequence, the Tt of an ELR sequence based on (VPGXG)_n_ can be controlled and fitted to the desired value (within a certain range) by varying the amino acid X at the fourth position (when X is a hydrophobic amino acid, the Tt decreases, and when it is hydrophilic the Tt increases) (Ribeiro et al., 2009). Although the pentapeptide (VPGXG) itself does not confer any biofunctionality on the ELR, recombinant technologies and modular design strategies allow the inclusion of functional epitopes, such as cell-adhesion domains (i.e., RGD) (Nicol et al., 1992; Urry et al., 1998), degradation sequences (i.e., protease-sensitive domains) (Alix, 2001; Girotti et al., 2004), sequences that promote the inclusion of temperature-related or biological triggers (Ulbrich et al., 1982; Miyata et al., 1999) and cross-linking motifs, in specific positions of the ELR backbone. This cross-linking can be of either a physical or a covalent nature, and the relevant mechanisms may involve ionic and hydrophobic interactions or the chemical reaction of complementary groups (Campoccia et al., 1998; Prestwich et al., 1998; Saha et al., 2020).

Such stimuli-responsive behavior, together with the proven biocompatibility (Urry et al., 1991) and ability to strictly control the molecular composition, and the fact that they can be functionalized, have positioned these ELRs as outstanding candidates for numerous biomedical applications (Rodriguez-Cabello et al., 2010, 2011), particularly as regards nanofiber-based scaffolds. As shown below, ELRs have been widely used to obtain nanofibers using both approaches that take advantage of their molecular self-assembly ability and advanced polymer-processing technologies.

Summarizing, ELRs are versatile materials that can be formulated with a huge variety of designs at aminoacidic level and that can tune their macromolecular appearance, physical behavior and mechanical properties depending on the molecular architecture, functional groups and the presence or absence of a concrete stimuli. Thus, ELRs can be formulated and processed in many different ways to obtain a diversity of structures such as nanoparticles, fibers, microfibers or hydrogels depending on the application field. These application fields comprise a large spectrum of areas in which the ELRs can be used, from protein or drug purification to complex systems for drug or gene-delivery or tissue regeneration through custom-designed scaffolds. González de Torre et al. (2014) obtained ELRs click hydrogels with an elastic moduli in the range 1–10 kPa, which are of widespread interest in tissue engineering application as many native tissues have moduli in this range (∼100 Pa for very soft tissues as fat or brain and ∼ 10.000 Pa for muscle). The ELR hydrogels can be engineered with a set of specific self-assembling domains that induce a sequential gelation mechanism and yield to extrudable bioinks with high printability and stability (Salinas-Fernández et al., 2020). The resulting 3D printing scaffolds, which preserve the structure long-term with high shape fidelity, are excellent candidates for the development of constructs, biological tissues or microorgans for different biomedical applications such as regenerative medicine and the validation of new drugs and therapies (Salinas-Fernández et al., 2020). ELRs have been widely used to obtain nanofibers using both approaches that take advantage of their molecular self-assembly ability and advanced polymer-processing technologies. As an example, ELR microfibrous scaffolds with a fiber width of 1.11 ± 0.45 μm and tensile strength of 0.59 ± 0.08 MPa have been obtained (González de Torre et al., 2018), which perfectly fits the values reported in the literature for fibers used as engineered ECM and, thus, are suitable candidates for tissue engineering or regenerative medicine applications.

## Silk-Elastin-Like Recombinamers (SELRs) and Silk-Tropoelastin Biomaterials

Silk is an exceptional example of a fibrous protein found in nature (Ling et al., 2018). A variety of organisms, such as spiders and silkworms, produce an array of silk fibers that are used to build proteinaceous scaffolds for protective and hunting purposes. The most common silk sequences exceed 3000 amino acids and consist of the repetition of hydrophobic and hydrophilic blocks, thus conferring an intrinsic amphiphilicity on them. Representative models are the major ampullate spidroins (MaSp1 and MaSp2) of dragline spider silk (Gatesy et al., 2001; Motriuk-Smith et al., 2005) and the heavy chain of *Bombyx mori* (*B. mori*) silk fibroin (Xia et al., 2004). MaSp1 and MaSp2 proteins show a hydrophobic poly(GA) block, containing poly(A) domains, which are flanked by hydrophilic GGX (X = Y, L, Q) and GPGXX (X = Q, G, Y) repeats, respectively (Gaines and Marcotte, 2008). Whereas, the *B. mori* heavy chain comprises repeated GX (X = A, S, T, V) and GAGAGX (X = A, S, Y) domains interspersed by hydrophilic, aromatic and charged motifs (Zhou et al., 2001; Sehnal and Žurovec, 2004).

The combination of hydrophobic and hydrophilic blocks found in native silk proteins (Jin and Kaplan, 2003; Lu et al., 2012) results in the formation of micellar structures (Lin et al., 2017; Parent et al., 2018; Hu et al., 2020) which, by way of a coalescence process triggered by the pH change and shear stress suffered while exiting the spinning duct, evolve into aligned nanofibrils (Holland et al., 2012; Schwarze et al., 2013) comprising β-sheet secondary structures built by assembly of the recurrent poly(A), (GA), and (GAGAGS) regions (Hu et al., 2006; Lewis, 2006).

Recently, the role of the *B. mori* hexapeptide GAGAGS, which is the active motif involved in the formation of H-bonded β-sheet structures, has been investigated by Sun et al. in detail (Sun and Marelli, 2020). As reported by these authors, a multi-step self-assembly pathway is required to structure this disordered motif into β-sheeted nanofibrils. Self-assembly begins once the critical micelle concentration has been exceeded, which induces the formation of micelles that hide the hydrophobic chains from the aqueous phase and minimize the energy of the system. These structures then disassemble, leading to the formation of high energy transient conformations that are able to orderly arrange into secondary β-sheet structures. Morphogenic kinetics slows down at this stage, thus resulting in a progressive increase in the number of β-sheets and silk nanofibrils that branch out from the pre-formed fibers. As these events progress, larger, thicker and stiffer structures arise, thereby conferring remarkable mechanical properties on these silk-like fibers (Sun and Marelli, 2020).

Silk proteins reconstituted from native fibers offer a readily accessible stock with great potential for investigating advanced approaches. Reconstitution of the silk dope involves removal of the adhesive sericins found between the fibers, followed by treatment with lithium salts, which disrupts the H-bonded β-sheet structures, thus disassociating the proteins into individual entities (Heim et al., 2009). Despite the simplicity of this process, the regenerated silk fails to replicate the native properties of silk protein (Shao et al., 2003; Holland et al., 2007; Greving et al., 2010; Wang and Zhang, 2013). This has led to the development of optimized processing methods that are able to circumvent the limitations encountered (Zhou et al., 2018; Guo et al., 2020b; Rizzo et al., 2020).

Alternatively, the emergence of recombinant techniques has allowed the bioproduction and subsequent study of silk-like proteins in greater detail, further offering the possibility to develop hybrid proteinaceous designs containing alternative protein-based sequences, such as elastin (Cappello et al., 1990). This family of polypeptides, known as silk-elastin-like proteins (SELPs) or recombinamers (SELRs) (Fernández-Colino et al., 2014; Wang et al., 2014), encompasses the silk-like GAGAGS sequence found in the heavy chain of *B. mori* fibroin (Hu et al., 2020), and the consensus elastin-like pentapeptide VPGXG (Acosta et al., 2020a), which includes any amino acid in the fourth position except proline.

The presence of silk-like domains confers the engineered SELR with the ability to self-assemble into fibrillary structures via H-bonded nanocrystalline β-sheet structures (Sun and Marelli, 2020). As such, fibrillary morphogenesis, which is not a prevailing feature of the ELRs (Misbah et al., 2015) but has been successfully imitated by the use of electrospinning techniques (Humenik et al., 2018), can be genetically encoded into the SELR constructs, thus conferring the inherent ability to form fibers comprising bundles of fibrils (Tarakanova and Buehler, 2012). In addition, molecular modeling has demonstrated that the thermoresponsive behavior of the elastin-like sequences is preserved in the SELR and favors the silk β-sheet assembly process (Tarakanova et al., 2017). Increasing the temperature induces the phase transition of the elastin-like domains, which tighten and hydrophobically fold the polypeptide backbone, thereby reducing the distance between the proteinaceous chains while fostering the formation of intramolecular H-bonds. At a molecular level, this rearrangement results in a significant shrinkage of the structure, thus enhancing the mechanical properties of the SELR in its fibrillary state.

Macroscopic evaluation of early silk-elastin-like designs revealed fibrous sponge-like structures typical of silk fibroin (Cappello et al., 1990; Dinner et al., 2000). This observation led to research focussing on understanding the role of elastin and silk-like domains in this fibrillary self-assembly. As reported by Hwang et al., when included as the guest residues in the elastin counterpart, positively charged lysine groups influence the polypeptide arrangement over mica surfaces (Hwang et al., 2009). Depending in the ionic strength, either fibrillary or spherical assemblies aggregate over the substrate. Thus, a weak ionic strength favors an ion-pairing interaction between the positively charged SELR and the negatively charged mica surface, thus providing nucleating sites that lead to the nanofibers. In contrast, an increase in salt concentration hinders this event, thereby directing the self-assembly process toward globular structures instead. Mica substrates, in combination with atomic force microscopy (AFM), can be exploited both to trigger the nucleation and to guide the direction of growth of SELR nanofibers via a bottom-up approach (Chang et al., 2011). Contacting the surface with the AFM tip initiates the nucleation of nanofibers which, given the finely applied pressure, elongate perpendicular to the scanning direction at a fast rate. This morphogenic pathway relies on events occurring at a molecular level. As described, the tip acts by stretching the stabilized and kinetically trapped SELR strands in the β-sheet, thus helping them to acquire transient conformations (Dinner et al., 2000) and therefore surpassing the high energetic barrier required to trigger the nucleation of fibrils (Varongchayakul et al., 2013). The growth stage likely continues with the self-assembly of SELRs into β-sheet structures parallel to the mica surface and perpendicular to the fibril axis drawn by the AFM tip.

Golinska et al. (2013) explored use of the octapeptide GAGAGAGE silk-like domain (Krejchi et al., 1994) to build pH-responsive SELR designs. Upon neutralization of the glutamic acid residue (Martens et al., 2009), this sequence self-assembles into β-roll assemblies (Yoder and Jurnak, 1995) from which fibrillary structures arise (Topilina et al., 2006; Werten et al., 2008). As predicted by molecular dynamics simulations, the slight differences between β-roll and β-sheet structures results in less-crystalline assemblies, and therefore semi-flexible SELR fibrils (Schor et al., 2009). On the other hand, elastin-like domains confer thermoresponsiveness on the construct. Increasing the temperature increases the number of nuclei as well as the fibril growth rate, with the morphological features remaining unaltered. However, the fibrillary structures progressively aggregate with time, undergoing an irreversible coalescence accompanied by a decrease in fiber length. Both the octapeptide GAGAGAGE and the hexapeptide GAGAGS can self-assemble into β-roll structures upon flanking four consecutive silk-like domains with lysine-rich hydrophilic elastin-like blocks in a core-sheath fashion (Zeng et al., 2014a). As reported by these authors, the presence of four consecutive silk-like domains is key to obtaining the β-roll assembly and the formation of nanofibrillar structures. Constructs containing three or fewer silk-like hexapeptides evolve into globular aggregates, whereas assembly is promoted above this threshold, thus resulting in a higher number of shorter fibrils. Switching from positively (lysine-rich) to negatively charged (glutamic acid-rich) elastin-like domains further affect the self-assembly process, via electrostatic interactions, changing the morphogenic pathway to cylindrical aggregates. Hydrophobic interactions also play a key role in the evolution of the structures formed leading, above the T_*t*_ of the elastin blocks, to the stacking of β-rolls and lateral coalescence of nanofibers over time.

Tuning the ratio of silk-to-elastin blocks further regulates the SELR self-assembly, providing access, irreversibly and reversibly, to fibrillar and micellar structures, respectively (Xia et al., 2011). As evaluated by circular dichroism (CD), an increase in the silk ratio favors β-sheet self-assembly via H-bonds, thus increasing the irreversible character of the SELR structures formed with time. Similarly, UV spectrometry suggests that an increase in the elastin ratio leads to the reversible formation of aggregates in solution that strongly absorb at 300 nm. The morphology of the structures obtained, as characterized by AFM, confirmed self-assembly of the elastin and silk-like blocks to form micellar to fibrillary structures in the micrometer range. Replica exchange molecular dynamics (REMD) and coarse-grained simulations also allow the effect of the silk-to-elastin ratio on the SELR interactions and fiber assembly to be investigated and predicted on a molecular scale. As described in the literature (Roberts et al., 2018), a high elastin content favors the structural compaction and H-bonding interaction at temperatures above the T_t_, thereby reducing the exposed area while decreasing the diameter of the SELR fibers, whereas a high silk content promotes the formation of β-sheet secondary structures that increase the fiber crystallinity and mechanical strength. Both proteinaceous blocks further act synergistically, thus improving the stability and strength of the assembled fibers.

Concentration is an extra parameter that governs the growth of SELR nanofibers. Thus, the micelle-like nanoparticles that arise during the early stages of self-assembly act by increasing the polypeptide density, thus prompting close approach of the silk-like domains, which subsequently self-assemble into nanofibers (Zeng et al., 2014b). Upon reaching this state, the soluble SELR begin to add, via diffusion pathways, to the formed structures, which evolve into different arrangements depending on the concentration. Thus, low SELR concentrations result in local gradients of polypeptide toward the tip of the fibers, whereas high concentrations result in the simultaneous and uniform formation of fibrils that branch out from the formed nanofibers, thus leading to interconnected networks over time. Concentration and the ratio of silk-to-elastin blocks can be exploited together to engineer template-induced fiber formation, that is, SELR constructs that are able to self-assemble into fibrils above a concentration threshold (Willems et al., 2019). Herein, the elastin-like content determines the disordered character of the proteinaceous backbone by limiting silk self-assembly and, thereby, the growth of fibrils at low concentrations. This can be counteracted by increasing the concentration and/or silk-like content, which favors the interaction and coalescence between aggregates, thereby opposing the amorphous character induced by the elastin-like domains and leading to long fibrils.

**FIGURE 3:**
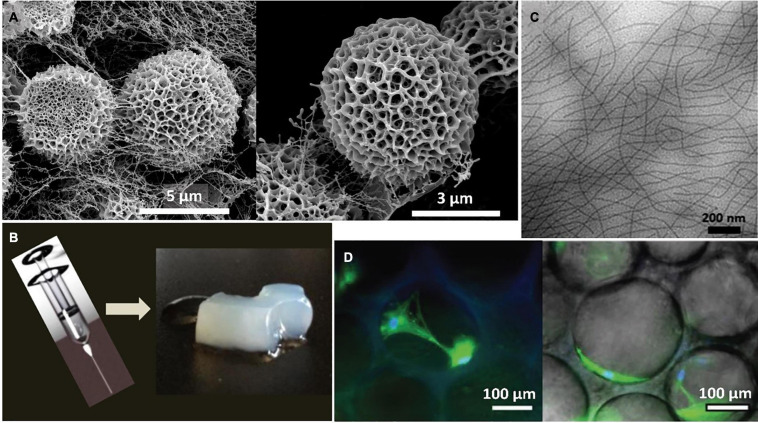
**(A)** Representative SEM images of the self-assembled biomorphs generated by EI-silk-VKV SELR from 2 mg mL-1 aqueous solutions after 48 h at 37°C reproduced with permission from (doi.org/10.1002/smll.202005191), copyright 2020. **(B)** Representative macroscopic images of the self-assembled hydrogel generated by (EIS)2 SELR from 150 mg mL-1 aqueous solutions after 60 days at 37°C reproduced with permission from (doi.org/10.1021/bm501051t2014), copyright 2014. **(C)** Representative TEM images of the self-assembled fibers generated by (EIS)2-(I5R)6 SELR from 3 mg mL-1 aqueous solutions after 48 h at 37°C reproduced with permission from (org/10.1021/acs.biomac.8b01211), copyright 2018. **(D)** Representative microscopic images of the self-assembled hydrogel generated by I_20_K_2_-RGD-K_2_S_6_I_20_ from 175 mg mL^–1^ aqueous solutions after 14 days post-seeding with human mesenchymal stem cells (hMSCs) at 37°C reproduced with permission from (doi.org/10.1002/smll.202001244), copyright 2020.”

As described by González et al., the intrinsically disordered and ordered character of SELRs can be exploited to design tailorable proteinaceous platforms for manufacturing morphologically rich fibrillary assemblies that mimic naturally occurring structures present in the natural type. These assemblies, known as biomorphs (Nakouzi and Steinbock, 2016), arise as a result of the effect of the hydrophobic, silk H-bonding and electrostatic interactions on the morphogenic pathway during the SELR desolvation stage, thus modulating and, eventually, arresting the self-assembly and transforming the transient structures into colporate biomorphic spheres (Figure 3A). The intrinsic disorder of the elastin-like sequence is required to structurally accommodate the three non-covalent interactions, whereas the silk-like domains kinetically trap the rearrangement of the SELR chains into the complex biomorphs by forming ordered β-sheet structures (González-Obeso et al., 2020).

SELRs have emerged as a versatile source for fibrillary scaffolds with potential applications in tissue engineering and the regenerative medicine field (Kapoor and Kundu, 2016; Aigner et al., 2018; Saric and Scheibel, 2019) as a result of their self-assembly properties and their biocompatible, biodegradable and highly tuneable character (Vepari and Kaplan, 2007; Guo et al., 2020a). For instance, injectable nanofibrillar hydrogels have been designed by combining silk and amphiphilic elastin-like blocks (Fernández-Colino et al., 2014). This configuration self-assembles, as a result of temperature, into micellar structures that evolve into nanofibrils with time. Each event is driven by a different block. Thus, the elastin-like repeats fold hydrophobically above the T_t_, triggering the formation of micelles and, thereby, spatial approximation of the silk-like blocks. This reorganization facilitates assembly of the silk domains into β-sheets, thus accelerating the formation of SELR fibrils, which evolve into a fibrous hydrogel, as can be seen in Figure 3B.

The synergic interplay between the silk and elastin-like blocks can be further exploited to freeze intermediate self-assembled states in time. This process, termed as pre-annealing treatment, involves incubating the SELR construct at different times while maintaining the temperature above the T_t_ of the elastin-like block (Cipriani et al., 2018). Optimization of this process allows advanced hydrophobically folded and β-sheeted self-assembled states to be selected (Figure 3C), thus conferring direct control over the mechanical properties and gelation time toward fibrous injectable SELR hydrogels. This approach has been explored for cartilage repair by manufacturing scaffolds including cell adhesive RGD sequences that help regenerate the hyaline cartilage in an *ex vivo* osteochondral model. The synergic ability of the elastin-like phase transition and the silk β-sheet self-assembling domains to capture the transient states arising during the formation of fibrillary SELR hydrogels has been recently investigated (Ibáñez-Fonseca et al., 2020). As observed, the different kinetics for the self-assembly of silk and elastin-like domains affect the structure achieved in a time-dependent manner. Initially, the phase-transition of the elastin-like blocks forces the molecule to undergo a rapid and entropically driven transition to a folded state, thus pushing the system to a new energy minimum. The silk-like domains then interact by slow formation of H-bonded β-sheets, thus creating an enthalpic energy barrier that prevents complete phase separation of the SELR from water. This metastable state evolves into a porous hydrogel that becomes kinetically trapped over time and can be seeded with human mesenchymal stem cells (hMSCs) (Figure 3D).

Recombinant tropoelastin has also been explored together with silk fibroin for the production of fibrillary structures. A strong complementarity exists between these two proteinaceous materials as a result of their hydrophobic repeats and their opposite net charge, i.e., +38 for tropoelastin and -36 for silk fibroin (Hu et al., 2013). The resulting ion-pairing interactions prompt their spontaneous aggregation which, under the influence of hydrophobic forces, drives the formation of micellar structures from which crystalline β-sheets emerge. Mono- and tri-layered membrane-like structures for treating age-related macular degeneration have been designed using silk-tropoelastin blends (Shadforth et al., 2015). β-sheet structures arise upon 12h of annealing at 60°C, providing a strength and elasticity similar to that of native Bruch’s membrane. In addition, both fabricated versions exhibit a cytocompatible character, and can be used as a potential vehicle for co-delivering retinal pigment epithelial cells and tropoelastin into the subretinal space.

## Electrospinning of Elastin-Based Materials

As mentioned previously, fibers can be obtained from biomaterials using different techniques. One of the most widely applied methods is electrospinning. This is not a new technique as the first reports date back to the early 20th century, although the electrostatic attraction of a liquid had already been observed by various scientists long before that (Tucker et al., 2012). Electrospinning technology has improved drastically since those early days, and the interest in the use of this methodology to create fibers is still increasing, as can be deduced from the large number of scientific papers published in this field every year. Electrospinning can be used to produce polymer fibers with diameters ranging from a few nanometers to several micrometers as a result of the electrical forces produced between two electrodes (Reneker and Yarin, 2008). This technique has become increasingly popular as a huge variety of polymers can be electrospun and because it offers the possibility to obtain fibers in the sub-micrometer range that can be applied in a wide variety of fields, such as filtration, optical electronics, defense, biotechnology and tissue engineering, amongst others (Bhardwaj and Kundu, 2010). Electrospinning is governed by several factors that require fine tuning to obtain fibers with the desired dimensions and properties. Briefly, these factors are applied voltage, flow rate, types of collector, distance between tip and collector, and ambient parameters such temperature and humidity (Reneker and Yarin, 2008; Bhardwaj and Kundu, 2010; Xue et al., 2017, 2019). The paramount importance of these parameters, and how to tune them to obtain the desired properties for the fibers, is beyond the scope of this review. Moreover, other excellent reviews focusing on the importance of these electrospinning parameters can be found in the literature (Reneker and Yarin, 2008; Islam et al., 2019; Xue et al., 2019).

This review is focused on the production of nano- and microfibers from elastin-based materials, especially materials made from recombinant elastin and tropoelastin, both of which have been electrospun under different conditions to obtain a wide variety of fibers for use in several biomedical applications, as will be discussed below.

## Electrospun Tropoelastin-Based Fibers

As is also the case for many other materials of biological origin, tropoelastin is suitable for electrospinning, typically after dissolution in a low boiling point solvent. Electrospun tropoelastin fibers can be obtained from nano to micron scale and need to be crosslinked after deposition. The resulting scaffolds must be crosslinked prior to use as platforms for tissue regeneration. The crosslinking agent is commonly glutaraldehyde (GA), which can be directly applied in solution or as fume from a 25% aqueous solution. Although hexamethylene diisocyanate (HMDI) has also been used to crosslink these electrospun scaffolds, it has to be carefully washed out to avoid undesirable cytotoxic effects (Nivison-Smith et al., 2010). After crosslinking, these scaffolds are extremely stable, maintaining their structural integrity for more than 180 days under physiological conditions (Almine et al., 2010). The fiber size, mechanical properties of the scaffolds, and their porosity can be precisely controlled by varying the aforementioned electrospinning parameters (Rnjak-Kovacina et al., 2011, 2012). In this way, highly elastic fibers with a Young’s modulus of 265 kPa on randomly aligned fibers, and 111 kPa on aligned ones, while maintaining an ultimate stress (116 kPa) and strain of about 1.5%, have been obtained (Nivison-Smith and Weiss, 2011). Electrospun elastin fibers exhibit a characteristic thin ribbon-like morphology, with diameters ranging from 0.9 to 5.5 μm for tropoelastin and 0.6 to 3.6 μm for α-elastin (Wise et al., 2009). Tropoelastin-based scaffolds exhibit good fibroblast colonization, producing their own extracellular matrix and even obtaining good vascularization in the surrounding tissues as a result of an optimal *in vivo* interaction with the adjacent native tissues (Rnjak et al., 2009; Rnjak-Kovacina et al., 2011). These electrospun tropoelastin-based scaffolds palliate one of the greatest problems that collagen, as a biomaterial, usually exhibits, namely contraction of the scaffold after cell colonization, which in some cases is over 50% of the original size of electrospun collagen scaffolds used in wound healing (Powell and Boyce, 2009) compared with 30% for electrospun tropoelastin scaffolds. These tropoelastin scaffolds have been shown to be cytocompatible with primary human elastic-tissue derived cells such as human fibroblasts (Fb), human umbilical vein endothelial cells (HUVECs) and human coronary artery smooth muscle cells (HCASMCs). Moreover, in the case of human adipose-derived stem cells (HADSCs), the use of electrospun tropoelastin scaffolds in the field of wound healing has been shown to increase the speed of wound closure as well as the epithelial thickness when compared with the untreated controls (Machula et al., 2014). Electrospun tropoelastin scaffolds have been used as a vehicle to deliver stem cells as they mimic the biological and mechanical features of the native extracellular matrix, and electrospun scaffolds loaded with adipose-derived stem cells help the wound-healing process *in vivo* due to their low immunogenicity (Machula et al., 2014).

Another important field in which electrospun tropoelastin scaffolds have been applied is the development of cardiovascular grafts. Thus, McKenna et al. developed a tubular vascular scaffold from tropoelastin with mechanical properties similar to those of native vessels, although the mechanical strength of these scaffolds made them unsuitable for implantation (McKenna et al., 2012). To overcome this problem, tropoelastin-based scaffolds can be co-electrospun with other natural or synthetic polymers (Heydarkhan-Hagvall et al., 2008; McClure et al., 2009, 2012; Zhang X. et al., 2009; Zhang et al., 2010; Rnjak-Kovacina et al., 2012) to create new hybrid materials with improved properties (Lee et al., 2007; Zhang et al., 2007; Han et al., 2011). Such hybrid materials have mainly been used to develop vascular substitutes by electrospinning composite solutions onto a rotating mandrel that acts a cylindrical collector in order to obtain tubular scaffolds. Collagen, PDO (polydioxanone), PLGA (poly(lactide-co-glycolide)), gelatin, PLLA (poly(L-lactide) acid), PLCL (poly(lactide-co-caprolactone)), and PCL (polycaprolactone), amongst others, are the polymers most commonly used in combination with tropoelastin to create these tubular structures (Li et al., 2005, 2006; Sell et al., 2006; Lee et al., 2007; McClure et al., 2010). These co-electrospun scaffolds are intended to mimic both the natural shape of the blood vessels and the internal structure, while maintaining the mechanical properties to withstand the pressure and pulsation of the blood stream (Boland et al., 2004). Approaches based on multilayered electrospun scaffolds have been explored to that end. For instance, a tri-layer scaffold based on collagen I and tropoelastin as biomaterials was investigated by Boland et al. This scaffold had an outer electrospun layer comprising tropoelastin and collagen in a proportion of 20:80 and an internal layer with a proportion of 70:30 (tropoelastin: collagen). Fibroblasts were seeded in the outer layer and smooth muscle cells and endothelial cells were seeded in the inner part of the construct. A layer of smooth muscle cells was added between these two layers to create the third layer of the scaffold. More tri-layered tubular electrospun scaffolds with different compositions and structures have been explored, for instance a synthetic vascular graft comprising an intima layer made exclusively of electrospun tropoelastin and PCL and an adventitia layer made of collagen and a PCL, with the media comprising a mixture of tropoelastin collagen and PCL, was created by McClure et al. (2010, 2012). Similarly, Wise et al. developed a bilayer tubular scaffold based on the sequential deposition of electrospun tropoelastin fibers followed by a mixture of tropoelastin and PCL (80:20) onto a rotating mandrel then crosslinked with glutaraldehyde vapor (Wise et al., 2011). Sequential deposition of electrospun layers of tropoelastin, gelatin and polyglyconate (Thomas et al., 2007), or even more complex mixtures such as tropoelastin, gelatin, poliglecaprone and PCL (Zhang X. et al., 2009), has been used to create these artificial vascular conduits. All these approaches show the extreme versatility of this technique and the almost infinite possibilities for creating mixtures of natural and synthetic polymers with tropoelastin in order to combine the outstanding biocompatibility of tropoelastin with the mechanical properties provided by the other polymers.

Although the vascular field is perhaps the principal target of electrospun tropoelastin-based composite scaffolds, other applications in tissue engineering have been explored. Briefly, Swindle-Reilly et al. created electrospun scaffolds from elastin and PCL with fiber diameters ranging from 400 nm to 1 mm for peripheral nerve regeneration. This scaffold promoted the adhesion and alignment growth of neurites in the direction of the aligned nanofibers (Swindle-Reilly et al., 2014). Similarly, the combination of collagen and elastin has been explored by Rnjak et al. to create electrospun scaffolds as artificial skin for wound healing. These scaffolds combine the best properties of both proteins, thus resulting in optimal scaffold handling and improving the elasticity with respect to collagen-only scaffolds while maintaining excellent cell attachment and proliferation with promising *in vivo* results (Rnjak et al., 2009; Rnjak-Kovacina et al., 2011, 2012).

### Electrospun ELR-Based Fibers

Elastin-like recombinamers are suitable materials for processing using different spinning techniques to obtain nano- and microfibers. For instance, fibers with diameters of a few tens of micrometers have been obtained using ELRs functionalized with silk fibroin (Qiu et al., 2009; Zeng et al., 2014a) using a wet spinning technique. Specifically, a solution of ELR-silk in methanol/water was extruded to obtain coagulated fibers, which were then dried and crosslinked. The diameter of the fibers can be controlled by varying the spinneret size and drawing the fibers during collection. Moreover, fiber patterning can be achieved by using mechanical stimuli to induce nucleation in a specific direction (Chang et al., 2011; Johnson et al., 2012). Some ELRs have been used alone as biomaterials for electrospinning in order to create fibrous scaffolds for tissue-engineering applications (Benitez et al., 2013). For instance, Putzu et al. (2016) developed tubular scaffolds of pure ELRs by using a rotating mandrel as collector electrode. The aim of this work was to create engineered vascular grafts, particularly with an inner layer containing a peptide sequence that promotes specific adhesion of endothelial cells, such as REDV (Arg-Glu-Asp-Val). This tetrapeptide is specifically recognized by the integrin a4b1 found in endothelial cells. The outer part of the scaffold contains the general cell-adhesion sequence RGD (Arg-Gly-Asp) to induce the recruitment of structural cells, such as fibroblasts and muscle cells. These scaffolds had to be stabilized by chemical crosslinking in a 0.1% solution of genipin in acetone, then soaked in phosphate medium to eliminate any harmful residues (Putzu et al., 2016). The same group continued investigating in the same direction to eliminate the use of chemical crosslinkers in order to ensure the total cytocompatibility of the engineered vascular grafts. To that end, they electrospun ELRs containing the GAGAGS (Gly-Ala-Gly-Ala-Gly-Ser) sequence from silk. This sequence spontaneously self-assembles into b-sheets stabilized by hydrogen bonding, thereby stabilizing the whole electrospun fiber. This approach was employed to cover metal stents and catheter balloons for cardiovascular applications (Putzu et al., 2019). Also following this approach, Machado et al. electrospun fibers from two different silk-elastin like polymers (SELPs) to create three-dimensional scaffolds for tissue-engineering applications. The fibers obtained exhibited diameters much smaller than obtained previously (100 ± 12 nm) and the typical ribbon-like morphology of the ELR-based electrospun fibers, thus suggesting that the scaffolds obtained could be suitable for the aforementioned applications (Machado et al., 2013).

**FIGURE 4:**
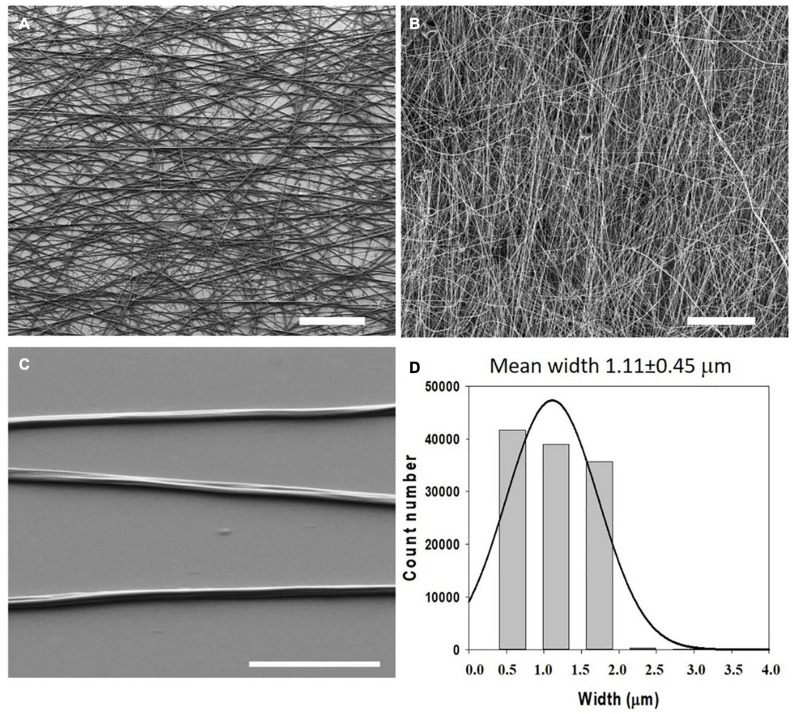
Morphology and dimensions of the first ELR-click fibers. SEM micrographs of ELR-click fibers at different magnifications. Scale bars: **(A,B)** 100 mm, **(C)** 10 mm. **(D)** Statistical distribution of the fiber widths was obtained using the DiameterJ plug-in of Fiji (ImageJ). Fitting to a Gaussian distribution (continuous line) gave a mean width of 1.11 ± 0.45 mm. Reproduced with permission from: (doi.org/10.1016/j.actbio.2018.03.027), copyright 2018.

**FIGURE 5:**
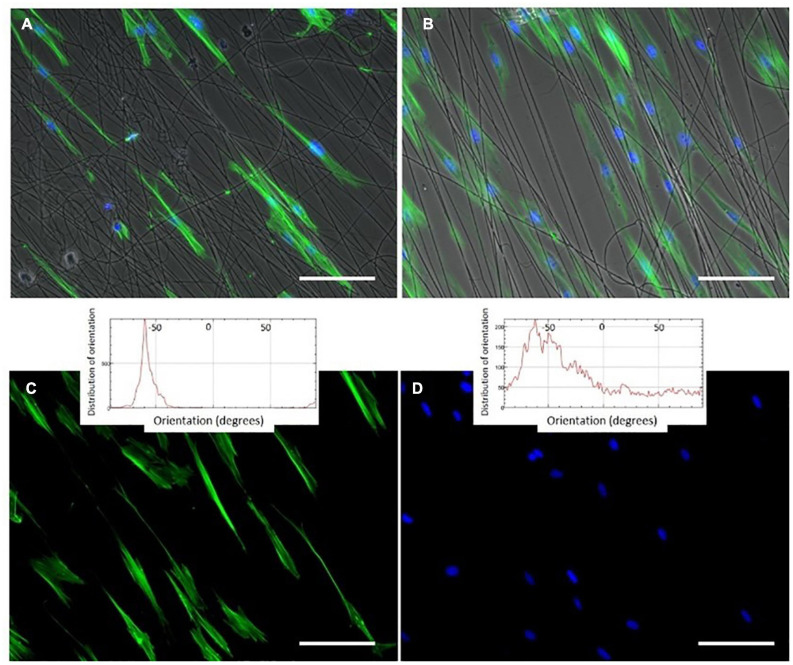
Phalloidin and DAPI staining (green (cytoplasm) and blue (nucleus)) of oriented HFF-1 cells on ELR-click fibers. All channels are merged in panels **(A,B)**, whereas images **(C,D)** show the green and blue channels for cytoplasm and nucleus staining, respectively, for a better visualization of the extended morphology of the aligned HFF-1 cells. The inserts represent the orientation data for the cytoplasm and nucleus, respectively. Scale bars: 50 mm. Reproduced with permission from: (doi.org/10.1016/j.actbio.2018.03.027), copyright 2018.

In 2018, Gonzalez de Torre et al. were able to create stable electrospun fibers that crosslinked during the flight of the jet from the needle to the collector by simultaneously electrospinning two complementary clickable ELRs. The scaffolds created in this way did not require any further crosslinking or stabilization step. The pure ELR-based fibers obtained exhibited a wrinkled morphology in both randomly oriented and aligned fibers (See Figures 4, 5). These ELR-based click fibers opened up the possibility to creating fibers from different ELRs bearing different bifunctionalities that can be selected depending on the final application. The scaffolds obtained offered a maximum strain of 247.5 ± 36.08% and a mean Young’s modulus of 1.73 ± 0.95 MPa while conserving the excellent cytocompatibility of the ELRs, as corroborated by *in vitro* experiments (González de Torre et al., 2018). Similarly, Fernandez-Colino et al. obtained stable ELR-based fibers with a mean diameter of 1.45 ± 0.32 μm from aqueous solutions (1:1 PBS/ethanol v/v). These fibers were collected on a rotating electrode, creating a small caliber tubular vascular graft.

Although recombinant tropoelastin and elastin have been blended with other natural and plastic polymers to obtain electrospun scaffolds, as described previously, ELRs have not been extensively processed together with other polymers to obtain electrospun scaffolds. However, they have been used to functionalize electrospun fibers from other synthetic polymers. Thus, Blit et al. (2012); Wong et al. (2013) modified the surface of electrospun polyurethanes with ELRs to increase the adherence of smooth muscle cells, finding a clear improvement in the cell covering of the substrate.

Table 1 has been included, in order to have a better perspective and a tentative comparation between electrospun fibers from elastin-based materials and other electrospun fibers from proteins used in the biomedical field. As can be seen, fibers from ELRs, SELPs and tropoelastin are in concordance with electrospun fibers from the other materials. Of course, in this table is only recorded a representative sample of all the works performed in this field.

**TABLE 1:** Properties and applications of different protein-based electrospun scaffolds.

Protein	Diameter (nm)	Tensile strength (MPa)	Young’s modulus (MPa)	Strain at break (%)	Applications
**Silk**	6.5–200	875–972	11–13	17–18	GBM, VTE, TE
**Collagen**	100–6000	8–12	262 ± 18	—	TE, GBM, WH, VTE
**Gelatin**	125–691	2.50	105	64	TE, WH
**ELR**	300–1500	0.59-43.3	1.73-1800	2.3-247.5	TE, GBM, WH
**Tropoelastin**	900–2700	13	289	—	TE
**SELP**	92–1593	30.8	880	7.9	GBM, TE

In order to clarify the table the references for each biomaterials hasn’t been included and can be found here: Silk references (Cunniff et al., 1994; Zarkoob et al., 2004; Wang et al., 2006; Alessandrino et al., 2008); Collagen references (Li et al., 2005; Venugopal et al., 2005; Casper et al., 2007; Yang et al., 2008); Gelatin references (Huang et al., 2004; Zhang et al., 2005; Songchotikunpan et al., 2008; Zhang S. et al., 2009); ELRs references (Huang et al., 2000; Nagapudi et al., 2002; Tan and Lim, 2006; González de Torre et al., 2018); Tropoelastin references (Li et al., 2005; Mithieux et al., 2009; Nivison-Smith et al., 2010); and SELP references (Ner et al., 2009; Machado et al., 2013).

## Zipper-ELR

**FIGURE 6:**
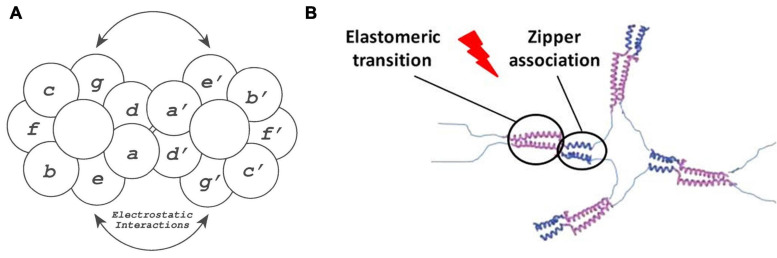
**(A)** Schematic representation of the zipper dimerization interface, where the 7 amino acids forming the alpha-helical turns are represented with letters. Herein ***a*** and *d*′ amino acids form the hydrophobic interactions, whereas ***e*** and ***g*′** amino acids form the electrostatic interactions reproduced with permission from (doi: 10.1101/gad.7.6.1047), copyright 1993. **(B)** Schematic representation of the elastin-like phase transition, represented in purple, and the dimerization of leucine zipper domains, represented in blue, in the formation of a ELR hydrogel reproduced with permission from (doi: 10.1021/acs.biomac.5b01103), copyright 2015.

Basic mammalian leucine zipper (B-ZIP) proteins, which have been reported to complex with sequence-specific double-stranded DNA (Vinson et al., 1989), are able to dimerize as a result of the interplay between the two amphipathic alpha-helices present in the leucine zipper structure, which create a stable interhelical salt bridge. The B-ZIP motif is contained in the N-terminal half of two clusters of basic amino acids, while the C-terminal contains an amphipathic amino acid sequence of variable length with a leucine every seven positions. This amphipathic sequence is termed the “leucine zipper” (Landschulz et al., 1988) and regulates the homo- and heterodimerization of B-ZIP proteins. Herein, in order to generate a repeating helical dimerization interface, the alpha-helix overtwists from 3.6 to 3.5 amino acids per turn, allowing the structure to repeat after 7 amino acids (a heptad repeat) or two alpha-helical turns. This coiled-coil structure is described in terms of the nomenclature of seven unique amino acid positions (***a, b, c, d, e, f, and g***) (Hodges et al., 1973), with the ***a*** and ***d*** residues typically being hydrophobic and being located in the “knobs and holes” pattern (predicted by Crick, 1953) along the dimerization interface of the alpha-helix. These amino acids interact with the complementary ***a*′** and ***d*′** amino acid positions of the opposite monomer (refers to the second alpha-helix in the dimer), thus providing the hydrophobic core essential for dimer stability (Thompson et al., 1993). In contrast, the ***g*** and ***e*** positions are typically charged amino acids (Cohen and Parry, 1990; Vinson et al., 1993) which, when forming attractive or repulsive interhelical electrostatic interactions between ***g*** and ***e*′**, referred to as i + 5 and i + 2 salt bridges (Marqusee and Baldwin, 1987; Cohen and Parry, 1990), can regulate both homo- and heterodimerization (see Figure 6). Furthermore, the Van der Waal interactions between ***g*** and ***e*′** methylene groups and the underlying ***a*** and ***d*** residues contribute to stability. Human B-ZIP domains have been classified into 12 families depending on the amino acids present in the ***a, d*, *e*,** and ***g*** positions, which appear to be critical for leucine zipper dimerization specificity (oligomerization, dimerization stability, and dimerization specificity). These have given rise to 12 families of B-ZIP with different properties, those that favor homodimerization (PAR, CREB, Oasis, and ATF6), those that favor both homo- and heterodimerization (C/EBP, ATF4, ATF2, JUN, and the small MAFs) and those that favor heterodimerization (FOS, CNC, and large MAFs; Vinson et al., 2002).

The inclusion of folded heptad repeats in the above-mentioned helical conformation can lead to the formation of fibrillary nanostructures (Herrmann and Aebi, 2004; Ryadnov et al., 2008). As described, oppositely charged sticky ends can prompt longitudinal propagation of the two-stranded α-helical coiled-coil zippers, thus yielding micrometric fibers (Pandya et al., 2000; Papapostolou et al., 2007). Specifically, the lateral association driving fibrillogenesis arises from the regularly spaced and complementary regions which, when interconnected by weak electrostatic forces, can extend and stabilize into filamentous assemblies. Indeed, the presence of salts as well as substitution of the sticky ends for blunt tails can disrupt this morphogenic pathway, thus resulting in coiled-coil assemblies that are unable to associate longitudinally. The tetrameric acidic and basic leucine-zipper dendrimers designed by the Ghosh group (Zhou et al., 2004) are one example of molecules that can form monodisperse and fibrillar micrometric structures from zipper sequences. Upon combination of both complexes, the hydrophobic and hydrophilic, i.e., pH-sensitive, regions self-assemble, driving the formation of helical secondary structures. This process continues with the lateral and longitudinal fusion of the repeating units, thus initiating fibrillogenesis and propagating the growth of fibers width- and lengthways, respectively. The Rodriguez-Cabello group has described the molecular assembly of elastin-like recombinamers with the hepatic leukemia factor HLF B-ZIP domains from the PAR family in self-assembling, temperature-triggered systems (Figure 6B; Fernández-Colino et al., 2015; Salinas-Fernández et al., 2020). Herein, the leucine zipper domains form amphipathic alpha-helical structures that interact hydrophobically, thereby promoting association into a coiled-coil supramolecular structure. This structure provides initial stability to the formation of fibrous hydrogels and to the development of a sequential three-stage gelation bioink that demonstrated the viability and proliferation of HFF-1 cells within the printed fibers.

## Amp-ELRs

**FIGURE 7:**
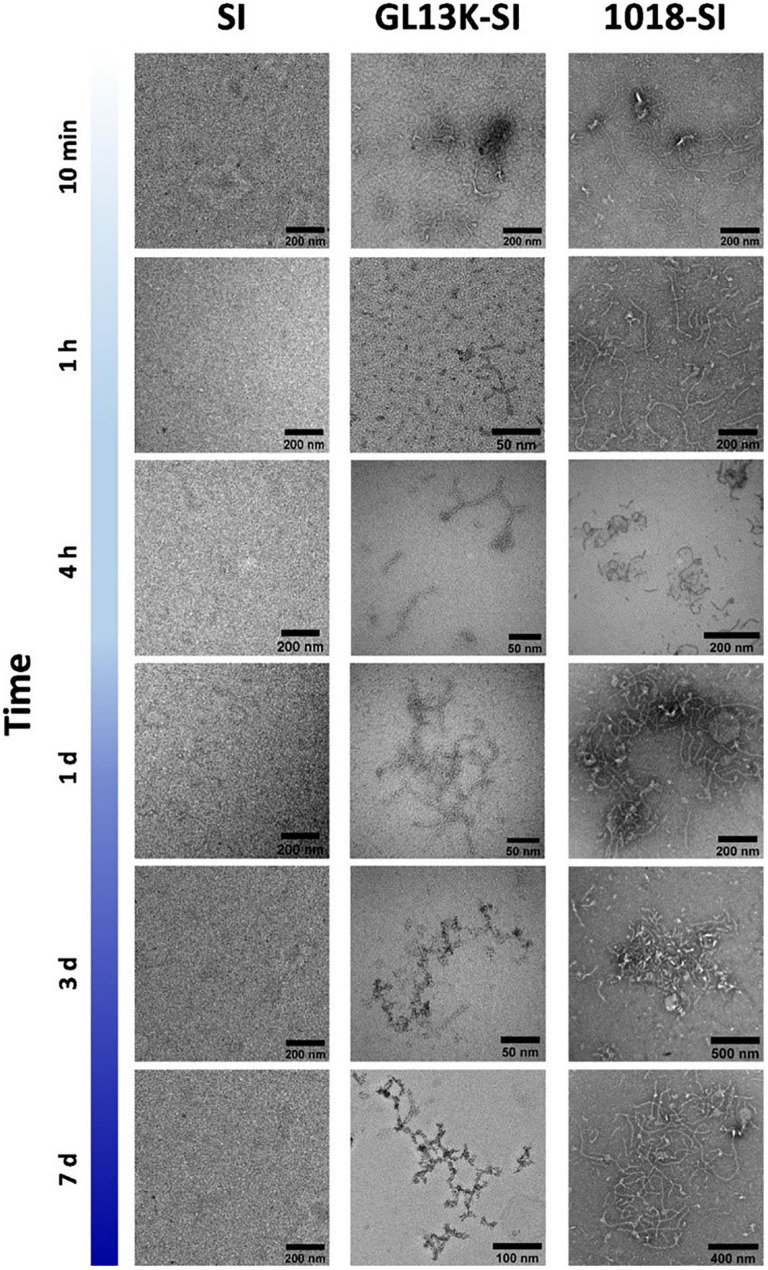
Representative TEM micrographs of small nanofibers formed by hybrid AMP-ELRs (GL13K-SI and 1018 SI), compared with control ELR (SI), after incubation at 5°C. Reproduced with permission from (doi: 10.1021/acs.biomac.0c00865), copyright 2020.

Natural and engineered antimicrobial peptides (AMPs) are generally short cationic peptides (10-50 amino acids). AMPs have attracted great interest in the treatment of bacterial infections, and their amphipathic nature also opens up the possibility of developing supramolecular nanostructures, such as fibers (Liu et al., 2013), nanoparticles (Goel et al., 2018), nanoribbons, or hydrogels (Veiga et al., 2012; Jiang et al., 2015; Baral et al., 2016; Nandi et al., 2017; Ye et al., 2019). For example, the AMPs GL13K and 1018 are able to self-assemble into stable secondary β-sheet structures (Haney et al., 2017; Ye et al., 2019), which can enable the organization of intrinsically disordered protein polymers (IDPPs) into hierarchical architectures. Acosta et al. described two hybrid recombinant AMP-ELRs using the AMPs GL13K and 1018 that allowed the development of small nanofibers based on a dual-assembly process. First, the interaction between AMP domains promotes the formation of nanofibers, which evolve into fibrillary aggregates in a synergic process driven by the thermoresponsive phase transition of the ELRs above the T_t_ (Acosta et al., 2020b) (See Figure 7). These authors also observed longer fibrils in the case of 1018 AMP-ELR, probably due to the higher tendency to create β-sheet conformations in solution at the same pH (Moussa et al., 2019). Furthermore, the tailorable functionality of these AMP-ELR nanofibers broadens their use for biomedical applications.

## Other ELR-Based Fibers

Incorporation of the laminin-1 sequence IKVAV into the ELR backbone has been explored to develop scaffolds capable of supporting neurotization (dos Santos et al., 2019). This sequence, which is responsible for promoting neuron cell attachment, migration and neurite outgrowth (Nomizu et al., 1995; Silva et al., 2004), confers fibrillary self-assembly properties on the elastin-based polypeptide chain, thus leading to irreversible association into fibrils. This self-assembly response and functionality, which is absent from the scrambled version including the VKAIV pentapeptide, was successfully exploited to manufacture polyethylene glycol crosslinked hydrogels that are able to support sensory neuron growth and promote neurite lengthening upon *in vivo* implantation in mice.

Finding inspiration in two hydrophobic motifs from tropoelastin (Wise et al., 2014), the Ohtsuki group designed an elastin-like pentapeptide with the ability to self-assemble into fibers (Le et al., 2017). The pentapeptide VGGVG spontaneously associates into secondary β-sheet structures, thus leading to the formation of beaded nanofibers. As electrostatic repulsion has been shown to negatively affect VGGVG fibrillogenesis, the inclusion of a positively charged tail, i.e., the KAAK tetramer, impaired the VGGVG self-assembly process, thus limiting the formation of β-sheets and slowing the kinetics toward thinner beaded nanofibers. Further addition of the cell-adhesive RGD domain conferred the intended bioactivity on the construct, although subsequent enhancement of the repulsive forces hindered the coalescence of nanoparticles on the forming nanofibers, thus decreasing the content of β-sheets. Subsequent studies revealed the dependence of self-assembly on the VGGVG content per molecule. As reported (Sugawara-Narutaki et al., 2019), an increase in the proportion of VGGVG favors the formation of β-sheet structures, thus leading to the morphogenic alignment and coalescence of nanoparticles into branched nanofibers, which laterally assemble to form bundles over time.

Alternative ELR designs, including the alanine-rich regions contained in the crosslinking domains of tropoelastin, i.e., (APGVGV)_x_ and (KAA(A)K)_n_ (Wise et al., 2014; Vrhovski and Weiss, 1998), have been described to undergo fibrillary morphogenesis (Djajamuliadi et al., 2020). Upon varying the temperature, this family of constructs undergoes a conformational transition from random coil to ordered secondary α-helix structures. This rearrangement, which was observed by solid-state nuclear magnetic resonance (SSNMR) spectroscopy in tandem with strategic isotopic labeling, correlates with the association of native elastin into fibers during elastogenesis (Muiznieks et al., 2003).

Oppositely charged ELRs have also been described to serve as building blocks for the manufacture of electrostatically driven fibrillary structures (Lee et al., 2001). Thus, fibrillogenesis occurs upon mixing a glutamic acid-rich with a lysine-rich ELR designed to contain an equal number and distribution of charged residues. Carbodiimide crosslinking of the assembled filamentous structures further leads to fibrous and transparent hydrogels that show a reversible anisotropic swelling behavior, becoming cloudy with increasing temperature.

## Conclusion

In this review, several procedures to obtain fibers and fibrous scaffolds from elastin-based materials have been described, from self-assembly processes to electrospinning. ELRs offer the possibility to modulate or adjust their structure from the designing stage which means the possibility to create new ELRs with specific properties, focused, in this case, on the formation of nanofibers. This is a huge advantage because we can obtain polymers with unique properties, but this requires designing, produce and purify new polymers that could not be an easy task and a deep molecular knowledge of the ELRs to be designed.

Although electrospinning is not a cutting-edge technology to obtain fibers and finally fibrous scaffolds to better mimic the microarchitecture of the ECM to be used in tissue engineering, it is a very versatile technique that can be used with a huge range of materials and concretely with elastin-based materials. Of course, some weaknesses can be found in this way to obtain fibers, and concretely fibers form elastin-based materials, as for instance a precise control of the spatial disposition of the nano or microfibers obtained, or the fact that many parameters (temperature, humidity, voltage, distance, and others) can affect to the quality of the fibers, but once the process is optimized the reproducibility obtaining electrospun fibrous scaffolds is quite high. Moreover, the use of potentially harmful solvent has been already avoided as was described in the previous pages. It is true that perhaps the 3D printing technologies could in a future substitute the use of electrospun scaffolds but the fiber dimensions and versatility in the use of biomaterials that electrospinning already offers to material researchers are still far from the reach of 3D printing technology and the use of it, is still a beautiful promise, at least at these levels. Therefore, electrospinning is still a technique that will continue evolving and used for the obtention of fibrous scaffolds.

## Author Contributions

IG, IM, MG-P, and FG-P participated in writing tasks. IG coordinated the writing tasks. JR-C directed, supervised, coordinated, and corrected the review. All authors contributed to the article and approved the submitted version.

## Conflict of Interest

The authors declare that the research was conducted in the absence of any commercial or financial relationships that could be construed as a potential conflict of interest.
